# Childhood Obesity and Maternal Personality Traits: A New Point of View on Obesity Behavioural Aspects

**DOI:** 10.3390/pediatric13030063

**Published:** 2021-09-01

**Authors:** Francesco Precenzano, Daniela Smirni, Luigi Vetri, Pierluigi Marzuillo, Valentina Lanzara, Ilaria Bitetti, Margherita Siciliano, Emanuele Miraglia del Giudice, Maria Esposito, Nicola Santoro, Marco Carotenuto

**Affiliations:** 1 Clinic of Child and Adolescent Neuropsychiatry, Department of Mental Health, Physical and Preventive Medicine, Università degli Studi della Campania “Luigi Vanvitelli”, 81100 Caserta, Italy; f.precenzano@hotmail.it (F.P.); luigi.vetri@unicampania.it (L.V.); valelanza87@hotmail.it (V.L.); ilaria.bitetti@gmail.com (I.B.); margaret.sici@hotmail.it (M.S.); maria.esposito3@unicampania.it (M.E.); marco.carotenuto@unicampania.it (M.C.); 2 Department of Psychology, Educational Science and Human Movement, University of Palermo, 90128 Palermo, Italy; daniela.smirni@unipa.it; 3 Oasi Research Institute-IRCCS, 94018 Troina, Italy; 4 Department of Woman, Child and of General and Specialized Surgery, Università degli Studi della Campania “Luigi Vanvitelli”, 80100 Naples, Italy; emanuele.miraglia@unicampania.it; 5 Department of Pediatrics, Yale University School of Medicine, New Haven, CT 06510, USA; nicola.santoro@yale.edu; 6 Department of Medicine and Health Sciences, “V. Tiberio” University of Molise, 86100 Campobasso, Italy

**Keywords:** personality assessment, paediatric obesity, maternal personality, MMPI-2

## Abstract

The epidemic spread of childhood obesity in Western society has interested many researchers, who agree in defining it as a multifactorial disease in which not only eating habits and sedentary lifestyle play a role, but also genetic predisposition. The aim of this study was to analyze the personality profile of a group of mothers of children with obesity and to compare this profile to that of a group of mothers of children without obesity. A total of 258 mothers participated in the study (126 mothers of children with obesity and 132 mothers of children without obesity). Weight and height were measured and the body mass index was calculated. The Minnesota Multiphasic Personality Inventory second edition (MMPI-2), evaluating personality and psychological disorders, was used to evaluate the personality profile. The results suggested that mothers of children with obesity score higher than the mothers of children without obesity in all MMPI-2 subscales. In most of these subscales, the differences between the two groups of mothers were statistically significant and with a medium to high effect size. These data suggest a new perspective on childhood obesity, identifying it as a multifactorial pathology that requires a multimodal and multidisciplinary approach that also takes care of caregivers to ensure optimal therapeutic efficacy.

## 1. Introduction

The epidemic explosion of childhood obesity in Western society has focused researchers’ attention on its etiology [1]. Actually, obesity is considered as a multifactorial disease in which both genetic predisposition and sedentary behaviour and incorrect food habits play a role [2]. Although the role of parents’ behaviour in children and adolescents’ eating habits [3,4,5], as well as the role of the home environment [6] and social status [7], in the etiology or maintenance of childhood obesity has been extensively investigated, little attention has been focused on the role of parents’ psychological habits in childhood obesity development. According to Zeller et al., the psychosocial factors of parents and families of children with obesity presenting for treatment in a clinic setting are different from those of parents of lean children, and maternal distress and mealtime challenges contribute to children’s obesity or leanness [8], stressing the key role of maternal habits in the development of childhood obesity. Moreover, previous research has outlined the association between being overweight during childhood and mothers’ educational level, concluding for a possible inverse correlation between the level of mothers’ acculturation and the degree of fat accumulation of their children [9]. On the other hand, a cross-sectional cohort study conducted in 2010 on a group of adolescents with obesity and their parents has revealed that the maternal psychopathology could be the strongest risk factor associated with bulimic behaviors in adolescents affected by obesity [10]. These findings suggested a putative role for the maternal psychopathology in the development of eating behavior disorders at developmental age [10]. In this perspective, the aim of our study is to assess the personality profile of a group of mothers of children with obesity and to compare these results to those of a group of mothers of lean children.

## 2. Materials and Methods

### 2.1. Participants

Two hundred and fifty-eight women participated in the study. A first group of 126 mothers of children with obesity, called “with obesity”, was enrolled at a third-level Universitary Endocrinologic Center for Childhood Obesity of the Università degli Studi della Campania “Luigi Vanvitelli” between January 2008 and December 2013. A second group of 132 mothers of children without obesity, called “control”, was recruited in the Pediatric Unit in which the Endocrinologic Center for Childhood Obesity is a specific section of the clinic service, among the outpatients and inpatients for pediatric problems different from obesity or overweight (i.e., gastrointestinal, growth, problems, and so on).

For the recruitment, the weight and height of all participants were measured. Body mass index (BMI) was calculated for the mothers and the standard deviations scores (SDS) for BMI using the LMS method [11] were computed for children.

Inclusion criteria: mothers of typical development children with no comorbidities.

Exclusion criteria: mothers with overweight or obesity (BMI ≥ 25); mothers of children with other disorders associated with obesity and/or neurodevelopmental disorders.

### 2.2. Methods

Minnesota Multiphasic Personality Inventory, second edition (MMPI-2).

The mothers’ personality was evaluated through the Minnesota Multiphasic Personality Inventory, second edition (MMPI-2), filled out by each mother and used to diagnose personality and psychological disorders [12,13]. MMPI-2 was made up of 567 true–false items and takes approximately 1 to 2 h to complete. The main differences between the two mother groups were analysed taking into account the two main scales: Clinical Basic Scale and Content Scale. The Clinical Basic Scale is used to identify different psychotic conditions and consists of 10 items: hypochondriasis (Hs) (used to evaluate excessive health anxiety), depression (D) (used to evaluate depression, lack of aims and hope in the future, general dissatisfaction, and poor morale), hysteria (Hy) (used to identify hysteric reactions to life events), psychopathic deviate (Pd) (used to identify psychopathic traits such as social deviation, refusal of authority, and amorality and can be thought of as a sign of disobedience), masculinity/femininity (Mf) (used to identify homosexual tendencies, but was found to be highly ineffective; high scores on this scale relate to factors such as intelligence, socioeconomic status, and education; women normally score lower on this scale), paranoia (Pa) (used to identify patients with paranoid traits inpatients such as persecution and paranoid feelings, grandiosity, extreme sensitivity, and rigid attitudes), psychasthenia (Pt) (considered distinctive of obsessive-compulsive disorders), schizophrenia (Sc) (used to identify schizophrenic traits in patients, including abnormal perception, bizarre or unusual thoughts, alienation, poor social relations, impulse control issues, lack of interests, and sexual difficulties), hypomania (Ma) (used to identify hypomania traits such as high speed speech, elevated mood alternated to short depression periods, reduced motor activity, and flight of ideas), and social introversion (Si) (used to evaluate withdrawal, social inhibition, and lack of responsibility). Moreover, the Content Scale permits the rational identification of content areas, clarifying the Clinical Scale interpretation and providing information not directly covered by the Clinical Scale. The Content Scale consists of 15 items: anxiety (ANX), fears (Frs), obsessiveness (Obs), depression (Dep), health concerns (Hea), bizarre mentation (Biz), anger (Ang), cynicism (Cyn), antisocial practices (Asp), type A (Tpa indicates hypermotivated, work-centered, and irritable subjects), low self-esteem (Lse), social discomfort (Sod), family problems (Fam), work interference (Wrk), and negative treatment indicators (Trt). In our study, MMPI-2 profiles met all the following validity criteria: less than 30 item omissions, *L* scale ≤ 65, *F* scale ≤ 100, *K* scale ≤ 65, *F*(*b*) scale ≤ 100, VRIN *T* score ≤ 80, TRIN *T* score ≤ 80, and *F*-*K* raw score ≤ 11. Specifically, the L scale (uncommon virtues) was designed to detect the intentional under-reporting, claiming an unusual number of uncommon virtues, while the K scale detects unintentional under-reporting, claiming to be well adjusted and free of psychopathology. The VRIN scale (variable response inconsistency) is designed to detect random responding and the tem content either similar or opposite in content, and can aid in the interpretation of infrequency scales. The TRIN (true response inconsistency) is a scale designed to detect fixed responding (acquiescence or counter acquiescence) and can aid in detecting fixed responding, difficulty reading/comprehension, and oppositionality.

MMPI-2 was evaluated by a trained psychologist (MS) to assess the personality of mothers of children both with and without obesity.

### 2.3. Ethical Statement

The study was approved by our Ethical Committee at the Università degli Studi della Campania “Luigi Vanvitelli” (protocol number 834/2016) according to the standards of the Declaration of Helsinki. Written informed consent was obtained by all the mothers enrolled in the study.

### 2.4. Statistical Analysis

The independent *t*-test was used to compare age, BMI, and MMPI-2 clinical basic and content scales between patients with obesity and control groups.

Cohen’s d was calculated in order to assess the effect size. To reduce the chances of obtaining false–positive results (type I errors) when multiple pairwise tests were performed on a single set of data, Bonferroni corrections (Bonferroni type adjustment) were applied by dividing the *p*-value by the number of comparisons being made. A *p*-value < 0.05 was considered statistically significant. The analysis was performed with STATISTICA software 6.0 version.

## 3. Results

No significant differences were found between the mothers of two groups (mothers of children with obesity vs. mothers of control children) for age (37.09 ± 6.86 vs. 36.67 ± 4.23; *t*-test_256_ = 0.595; *p* = 0.552), BMI (22.58 ± 1.723 vs. 22.89 ± 1.252; *t*-test_256_ = 1.659; *p* = 0.098), and educational level (11.34 ± 4.42 vs. 10.89 ± 3.79; *t*-test_256_ = 0.879; *p* = 0.38).

Concerning the couple status, both the groups of mothers are represented by married women with stable and non-separated relationships. The economic level was identified as middle-class socioeconomic status (between class 2 or class 3—corresponding to 28,000–55,000 euros/year to 55,000–75,000 euros/year, respectively, according to the current Italian economic legislation parameters).

Concerning children, the mean age was 10.3 ± 2.94 years; 128 males (49.6%); and the BMI-SDS mean was 2.05 ± 0.7.

The children of mothers belonging to the group with obesity (n. 126) reported a BMI >95 centile and BMI-SDS mean of 2.95 ± 0.9, while the children of mothers of the control group (n. 132) reported a BMI <85 centile and BMI-SDS mean of 0.19 ± 0.2. As expected, comparing means of BMI-SDS in children with obesity to children without obesity, a significant difference emerged (*t*-test_256_ = 34.36; *p* < 0.0001).

Table 1 shows the differences and the effect size in Clinical Basic Scales among the two groups. No significant differences in psychopathic deviate and masculinity/femininity scales were found between the two groups.

Specifically, in the Clinical Basic Scale, the mothers of children with obesity show significant higher scores in depression (*t*-test_256_ = 4.658; *p* < 0.0001, Cohen’s *d* = 0.579), hysteria (*t*-test_256_ = 5.847; *p* < 0.0001, Cohen’s *d* = 0.725), psychasthenia (*t*-test_256_ = 6.028; *p* < 0.0001, Cohen’s *d* = 0.769), hypomania (*t*-test_256_ = 3.404; *p* = 0.0008, Cohen’s *d* = 0.422), and social introversion (*t*-test_256_ = 5.723; *p* < 0.0001, Cohen’s *d* = 0.711) scales than the mothers of lean children, as well as in hypochondriasis (*t*-test_256_ = 8.462 *p* < 0.0001, Cohen’s *d* = 1.05), paranoia (*t*-test_256_ = 9.331; *p* <0.0001, Cohen’s *d* = 1.157), and schizophrenia (*t*-test_256_ = 7.120; *p* < 0.0001, Cohen’s *d* = 0.882) subscales.

In the Content Scale (Table 2), the mothers of children affected by obesity show no pathological scores in all scales even if they showed statistically significant higher scores in anxiety (*t*-test_256_ = 7.260; *p* < 0.0001, Cohen’s *d* = 0.900), fears (*t*-test_256_ = 7.236; *p* < 0.0001, Cohen’s *d* = 0.899), obsessiveness (*t*-test_256_ = 3.641; *p* = 0.0003, Cohen’s *d* = 0.452), depression (*t*-test_256_ = 5.510; *p* < 0.0001, Cohen’s *d* = 0.682), health concerns (*t*-test_256_ = 11.243; *p* < 0.0001, Cohen’s *d* = 1.395), bizarre mentation (*t*-test_256_ = 5.352; *p* < 0.0001, Cohen’s *d* = 0.663), low self-esteem (*t*-test_256_ = 6.630; *p* < 0.0001, Cohen’s *d* = 0.822), social discomfort (*t*-test_256_ = 3.571; *p* = 0.0004, Cohen’s *d* = 0.444), family problems (*t*-test_256_ = 4.566; *p* < 0.0001, Cohen’s *d* = 0.566), work interference (*t*-test_256_ = 5.240; *p* < 0.0001, Cohen’s *d* = 0.648), and negative treatment indicators (*t*-test_256_ = 5.539; *p* < 0.0001, Cohen’s *d* = 0.687) than the control mothers.

According to Sawilowsky’s new effect size rule of thumb, a Cohen’s d (0.01) = very small, d (0.2) = small, d (0.5) = medium, d (0.8) = large, d (1.2) = very large, and d (2.0) = huge [14].

## 4. Discussion

The mothers of the two groups were comparable in demographics. However, mothers of children affected by obesity seem to present peculiar personality traits compared with mothers of children without obesity, although they have to be considered as not pathological. Considering the effect size analysis, all the scores among the Clinical Basic Scale have a medium–large value, except for the hypomania subscale. Similarly, in the Content Scale, we reported a medium effect size for each subscale, except for anger, obsessiveness, cynicism, antisocial practices, and social discomfort subscales, which have small values. Interestingly, the health concerns subscale has a very large effect size, suggesting the real and relevant worries regarding the health conditions, probably including their own children. Although this subscale shows the strongest result in the analysis of the effect size, it is clearly to be considered as a possible datum perhaps common to the mothers of children with obesity or to those with children suffering from chronic pathology. However, it is mandatory to underline that the extrapolations that can be made on the present findings about the personality assessment must be considered in any case as interpretations and never absolute.

These findings may be intended to support the close relationship between obesity and psychological profiles, which is well known both in adulthood [15,16,17,18,19] and childhood [20], as well as the importance of self-esteem, body image, and social mobility impairment in children with obesity [21,22]. The neuropsychological and emotional impairment seems to be closely related to the overweight characteristic, independent of age and gender.

Moreover, the relationship between body size and depressive symptoms in young adolescents is yet to be clarified, stressing the role of BMI variations (higher or lower than the norm) in the pathogenesis of depressive symptoms [23]. Some studies about the role of maternal behaviors in the development and maintenance of childhood obesity have been conducted [3,4,5,9,10], but no conclusive data were collected about the personality of the mothers of children with obesity. On the contrary, our results show that the mothers of children affected by obesity have a peculiar personality profile, not necessarily to be considered as pathological. Sutin and Terraciano in their research analysed the association between mother and child personality and feeding strategies and a child’s BMI. Interestingly, they found that children with obesity had mothers with low conscientiousness personality traits. Conscientiousness was significantly related to less use of restriction and lower pressure to eat and more use of monitoring feeding strategies. The authors suggested that restriction and pressure to eat mediated the association between mother conscientiousness and child BMI [24]. Our data show that the personality of the mothers of children with obesity appears to be different compared with the personality of the mothers of children without obesity, suggesting a putative additional role of maternal psychological habits in childhood obesity development [25]. On the other hand, obesity is certainly a multifactorial pathology, and in no case can our data be identifiable as causative.

During childhood, eating behaviours should be established under the absolute maternal control to select what kind of foods the child should eat, which foods should be forbidden, when and where they should eat, as well as assessing the emotional colour of eating occasions [26]. Anyway, it is known that child-feeding practices may be one way in which a mother’s own concerns and insecurities surrounding eating may also manifest [27]. In this scenario, theoretically, the degree of maternal control exerted over the child-feeding structure could be a potential behavioural mechanism of attitudes and beliefs transmitted to children [28]. In this regard, in 2003, Trombini et al. found a significant prevalence of the insecure attachment style in a group of mothers of children with obesity compared with the control group of mothers of normal weight children [29]. These findings seem to be confirmed by our report of the higher scores in anxiety, obsessiveness, health concerns, bizarre mentation, low self-esteem, and social discomfort subscales among the mothers of subjects affected by obesity than ones of children without obesity. Moreover, youth self-report and mother-report of youths’ psychological difficulties were often most strongly associated with mothers’ level of psychological distress rather than with youth physical characteristics [30]. Jang et al., in their systematic review, showed how several types of parental stress are able to influence children’s weight. In particular, they found a positive relationship between parental general stress, parenting role stress, and child obesity [31,32]. In addition, the relationship between maternal psychopathological symptoms and later adolescent psychopathological symptoms was found to be partially mediated by maternal parenting self-esteem [29,30], impaired in mothers of children with obesity recruited in the present study.

Moreover, Trombini et al. discussed the psychiatric disorders among parents of patients with eating behaviour disturbances, highlighting that parents of patients with bulimic behaviours showed higher occurrences of paranoid and schizotypal personality styles [29], similar to our findings in mothers with obesity personality traits, although bulimic disorder and obesity are different clinical conditions.

In general, we have to specify and highlight that the terms personality profile, psychological habits, and attitudes cannot be considered as synonymous. In fact, personality profile may be considered as a method to show the results of psychological testing in graphic form, while psychological habits tend to correspond to a well-learned behavior or automatic sequence of behaviors that is relatively situation-specific and over time, and attitudes are a settled way of thinking or feeling about something. Therefore, these terms explore different dimensions of day-to-day life and cannot be used indifferently or to connote a pathological dimension of personality. In the present study, we used MMPI-2, which is a tool to assess the psychopathological aspects of adults’ personality in the hypothesis of finding psychopathological aspects, as part of the literature seemed to suggest.

This study is subject to some limitations. The results were obtained by administering a single test to the mothers and without a specific psychiatric evaluation. Moreover, MMPI-2 evaluation was used in order to reliably measure personality traits with the specific limit of emphasis in psychopathological aspects. Although the sample was compared with a large control group, the results are based on a relatively small number of participants and further studies would be needed to confirm the putative association between a peculiar personality profile in mothers and childhood obesity.

Despite these limitations, this study gives new insights into the clinical approach to childhood obesity and the personality profile of mothers of children with obesity.

## 5. Conclusions

To summarize, childhood obesity is complex disease and may considered as the final effect of interplaying between genetic and epigenetic factors. Moreover, among the many variables for the present research, the relationship between mother and child was chosen as its focus, taking into consideration the functioning of the mother’s personality as a putative relevant variable in this relationship.

Finally, our findings may be understood as limited to the evaluation of frankly psychopathological aspects that, indeed, appear absent in our sample of mothers of children with obesity.

In conclusion, this study suggests a different and new approach to childhood obesity. It would be a multifactorial pathology that requires a multimodal and multidisciplinary approach in which the need to take care of caregivers also emerges to ensure optimal therapeutic efficacy.

## Figures and Tables

**Table 1:** Mean differences in Basic Scale of the MMPI-2 test between the groups of mothers of children with and without obesity.

	with Obesity	Control	*t*-Test	*p*-Value	Cohen’s d
	*n* = 126	*n* = 132	df: 256		
Hypochondriasis	61.817 ± 10.915	51.788 ± 7.955	8.462	<0.0001	1.05
Depression	54.341 ± 8.801	49.508 ± 7.857	4.658	<0.0001	0.579
Hysteria	53.413 ± 10.544	46.750 ± 7.585	5.847	<0.0001	0.725
Psychopathic Deviate	55.849 ± 9.724	54.220 ± 4.882	1.712	NS	-
Masculinity/Femininity	53.754 ± 9.721	52.015 ± 9.942	1.420	NS	-
Paranoia	60.929 ± 12.830	48.182 ± 8.832	9.331	<0.0001	1.157
Psychasthenia	54.651 ± 11.349	47.038 ± 8.175	6.028	<0.0001	0.769
Schizophrenia	58.952 ± 9.613	51.909 ± 5.924	7.120	<0.0001	0.882
Hypomania	54.984 ± 12.817	50.038 ± 10.452	3.404	0.0008	0.422
Social introversion	54.929 ± 9.307	48.697 ± 8.170	5.723	<0.0001	0.711

Legend: The descriptive statistic was expressed as means and standard deviations (±) and differences of means (*t*-test) between the two groups: mothers of children with obesity (indicated as with obesity) vs. mothers of normal thin children (indicated as control). NS: not significant differences. df: degrees of freedom. Bonferroni corrections (*p* < 0.05/10 = 0.005).

**Table 2:** Mean differences in Content Scale of the MMPI-2 test between the mothers of children with and without obesity.

	with Obesity *n* = 126	Control *n* = 132	*t*-Test df: 256	*p*-Value	Cohen’s d
Anxiety	59.103 ± 11.467	50.091 ± 8.285	7.260	<0.0001	0.900
Fears	61.056 ± 10.305	52.530 ± 8.576	7.236	<0.0001	0.899
Obsessiveness	54.722 ± 10.989	50.174 ± 9.019	3.641	0.0003	0.452
Depression	54.913 ± 11.084	48.621 ± 6.86	5.510	<0.0001	0.682
Health Concerns	64.27 ± 10.642	51.371 ± 7.599	11.243	<0.0001	1.395
Bizarre mentation	59.063 ± 11.785	52.424 ± 7.83	5.352	<0.0001	0.663
Anger	52.651 ± 11.6	49.886 ± 8.315	2.208	0.0281	0.273
Cynicism	57.127 ± 11.102	53.576 ± 10.789	2.605	0.0097	0.324
Antisocial Practices	52.659 ± 9.377	49.78 ± 9.326	2.472	0.0141	0.307
Type A	53.833 ± 13.206	51.515 ± 9.789	1.607	NS	-
Low self-esteem	55.373 ± 9.373	48.712 ± 6.583	6.630	<0.0001	0.822
Social Discomfort	53.27 ± 9.019	49.356 ± 8.584	3.571	0.0004	0.444
Family Problems	52.294 ± 9.767	47.545 ± 6.726	4.566	<0.0001	0.566
Work interference	54.952 ± 10.988	49.205 ± 6.028	5.240	<0.0001	0.648
Negative treatment indicators	58.135 ± 11.067	51.598 ± 7.653	5.539	<0.0001	0.687

Legend: The descriptive statistic was expressed as means and standard deviations (±) and differences of means (*t*-test) between the two groups: mothers of children with obesity (indicated as with obesity) vs. mothers of normal thin children (indicated as control). NS: not significant differences. df: degrees of freedom. Bonferroni corrections (*p* < 0.05/15 = 0.002).

## Data Availability

Data supporting the reported results can be obtained on request.
